# Mobile Phone Based Clinical Microscopy for Global Health Applications

**DOI:** 10.1371/journal.pone.0006320

**Published:** 2009-07-22

**Authors:** David N. Breslauer, Robi N. Maamari, Neil A. Switz, Wilbur A. Lam, Daniel A. Fletcher

**Affiliations:** 1 UCSF/UC Berkeley Bioengineering Graduate Group; 2 Department of Bioengineering, University of California Berkeley, Berkeley, California, United States of America; 3 Biophysics Graduate Group, University of California Berkeley, Berkeley, California, United States of America; 4 Department of Pediatrics, University of California San Francisco, San Francisco, California, United States of America; McGill University, Canada

## Abstract

Light microscopy provides a simple, cost-effective, and vital method for the diagnosis and screening of hematologic and infectious diseases. In many regions of the world, however, the required equipment is either unavailable or insufficiently portable, and operators may not possess adequate training to make full use of the images obtained. Counterintuitively, these same regions are often well served by mobile phone networks, suggesting the possibility of leveraging portable, camera-enabled mobile phones for diagnostic imaging and telemedicine. Toward this end we have built a mobile phone-mounted light microscope and demonstrated its potential for clinical use by imaging *P. falciparum*-infected and sickle red blood cells in brightfield and *M. tuberculosis*-infected sputum samples in fluorescence with LED excitation. In all cases resolution exceeded that necessary to detect blood cell and microorganism morphology, and with the tuberculosis samples we took further advantage of the digitized images to demonstrate automated bacillus counting via image analysis software. We expect such a telemedicine system for global healthcare via mobile phone – offering inexpensive brightfield and fluorescence microscopy integrated with automated image analysis – to provide an important tool for disease diagnosis and screening, particularly in the developing world and rural areas where laboratory facilities are scarce but mobile phone infrastructure is extensive.

## Introduction

Light microscopy is an essential tool in modern healthcare. The advent of digital imaging has only enhanced this diagnostic role, as sample images are now frequently transferred among technologically-advanced hospitals for further consultation and evaluation, a role important enough that a medical communication standard (DICOM [1]) has been widely adopted for the handling of digital images. Unfortunately, much of the power of light microscopy, especially fluorescence imaging and the opportunity for remote consultation and electronic record keeping, remains inaccessible in rural and developing areas due to prohibitive equipment and training costs. This is especially problematic since the diagnosis, screening, and monitoring of treatment for many diseases and infections endemic to such areas – *e.g.* tuberculosis (TB), malaria, and sickle cell disease – depend on light microscopy as a screening tool or a definitive diagnostic test [2], [3], [4], [5], [6].

A recent convergence of technologies is making it possible to change the way microscopy is performed in developing countries. Given the ubiquity of mobile phone networks, the fact that many mobile phones are now equipped with digital cameras, the increase in computational power of mobile phones, and the advent of inexpensive high-power light emitting diodes (LEDs), we believe that these technologies can be combined to create an inexpensive and powerful tool for light (and especially fluorescence) microscopy in developing regions. While the concept and practice of telemedicine has existed for decades, it has only recently begun a shift to wireless platforms [7], [8], [9], and new avenues are now opening for developing mobile phone enabled medical technology [9], [10], [11]. An additional advantage to using a phone-based microscope is that mobile phones are essentially computers that can be used for digital image processing as well as electronic medical record keeping and communication.

Our initial device development efforts have been aimed at using the digital imaging capabilities, mobile connectivity, and computational power of a camera-enabled mobile phone to capture high-resolution microscopy images and perform subsequent image transmission or analysis. It has been previously demonstrated that a camera-enabled mobile phone can be used to capture images from the eyepiece of a standard microscope [11] and that microscopy images can be wirelessly transmitted for subsequent analysis [12]. However, our goal was to demonstrate the feasibility of creating an entirely integrated and portable mobile phone microscopy system. With the growing use of fluorescent stains in sample preparation to increase diagnostic sensitivity and specificity, we furthermore sought to incorporate fluorescent imaging capabilities into our mobile microscopy system and test the use of digital image processing for image analysis.

Here we report the development of a high-resolution microscope attachment for camera-enabled mobile phones that is capable of both brightfield and fluorescence imaging. We demonstrate the ability to use this system to capture brightfield digital color images of malaria parasites in thin and thick blood smears, sickled red blood cells in peripheral blood smears, and, using fluorescence, tuberculosis bacilli in Auramine O-stained sputum smears. Furthermore, we demonstrate the potential for improving diagnostic efficiency by using simple image processing software to label and count tuberculosis bacteria in a captured image, relieving healthcare workers of the time-consuming and error-prone task of counting by eye. We believe that by integrating these technologies, healthcare workers in remote regions equipped with microscopy-enabled mobile phones could take diagnostic images of patient samples (blood, sputum, etc.), perform on-board image analysis and/or wirelessly transmit those images off-site for medical record keeping, epidemic tracking, or further analysis by clinical experts.

## Results

### System Design and Characterization

**Figure 1:**
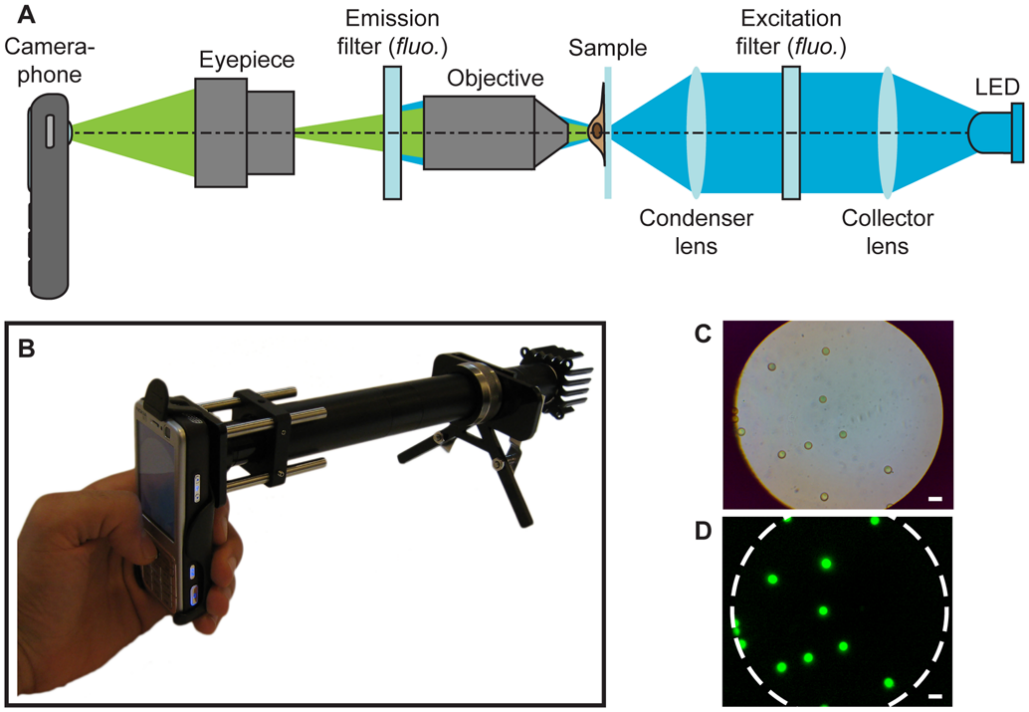
Mobile phone microscopy layout schematic, prototype, and sample images. (a) Mobile phone microscopy optical layout for fluorescence imaging. The same apparatus was used for brightfield imaging, with the filters and LED removed. Components only required for fluorescence imaging are indicated by “fluo.” Not to scale. (b) A current prototype, with filters and LED installed, capable of fluorescence imaging. The objective is not visible because it is contained within the optical tubing, and the sample is mounted adjacent to the metallic focusing knob. (c) Brightfield image of 6 µm fluorescent beads. (d) Fluorescent images of beads shown in (c). The field-of-view projected onto the camera phone CMOS is outlined. Scales bars are 10 µm.

Both the brightfield and fluorescence instruments are designed to work with a typical camera-enabled mobile phone (Figure 1a,b). The system uses standard, inexpensive microscope eyepieces and objectives; magnification and resolution can be adjusted by using different objectives. For this study, we used a 0.85 NA 60X Achromat objective and a 20X wide field microscope eyepiece, resulting in a system field-of-view of ∼180 µm diameter, an effective magnification onto the camera face of ∼28X, and a measured spatial resolution of ∼1.2 µm. The effective magnification figure requires care in interpretation as the image can take on greater magnification via digital enlargement. Resolution is a more fundamental parameter, and we estimated it to be ∼1.2 µm, based on the full width at half maximum (FWHM) of the system point-spread function (PSF, see Materials and Methods). This is a factor of three larger than the nominal Rayleigh resolution limit of 0.4 µm for the system, to be expected since the (purposefully inexpensive) objectives used are uncorrected for field curvature and other aberrations, reducing resolution away from the field radius of best focus. Imperfections and aberrations in the mobile phone lens will also contribute to the non-diffraction limited performance. Despite these limitations, the mobile phone camera was able to capture high-resolution images of blood and sputum samples useful for diagnosis.

Ambient light (without a condenser) was typically sufficient for brightfield imaging, but we also used a white LED for illumination in darker conditions. For fluorescence microscopy we utilized a simple and inexpensive trans-illumination geometry incorporating an LED excitation source and filters in the optical train (Figure 1a). High-power LEDs are now available in a wide range of emission bands, allowing for the matching of excitation wavelength with a variety of potential fluorophores. As others have also noted, the low cost, high robustness to mechanical shock and environmental conditions, low power requirements, ambient operating temperatures, and ∼50,000 hour lifetimes of LEDs make them particularly suitable for use in portable systems and systems designed for use in developing areas where replacement parts may be unavailable or unaffordable [2], [13], [14]. In our fluorescence system, illumination was provided by a high-power blue LED, emitting within the excitation range of the fluorescent Auramine O stain commonly used for detection of TB bacilli in sputum samples. Sensor integration time for the phone was unavailable, so the limiting system sensitivity could not be determined. Whereas an epi-illumination geometry is generally used to minimize background from the illumination source in fluorescence microscopy, we found that the Auramine O-stained TB fluorescence was more than sufficiently bright for bacillus identification using our trans-illumination geometry, which in turn reduces the complexity and cost of our system- an important consideration given the resource-poor settings where it could be of use.

### Brightfield Imaging of Malaria and Sickle Cell Anemia

**Figure 2:**
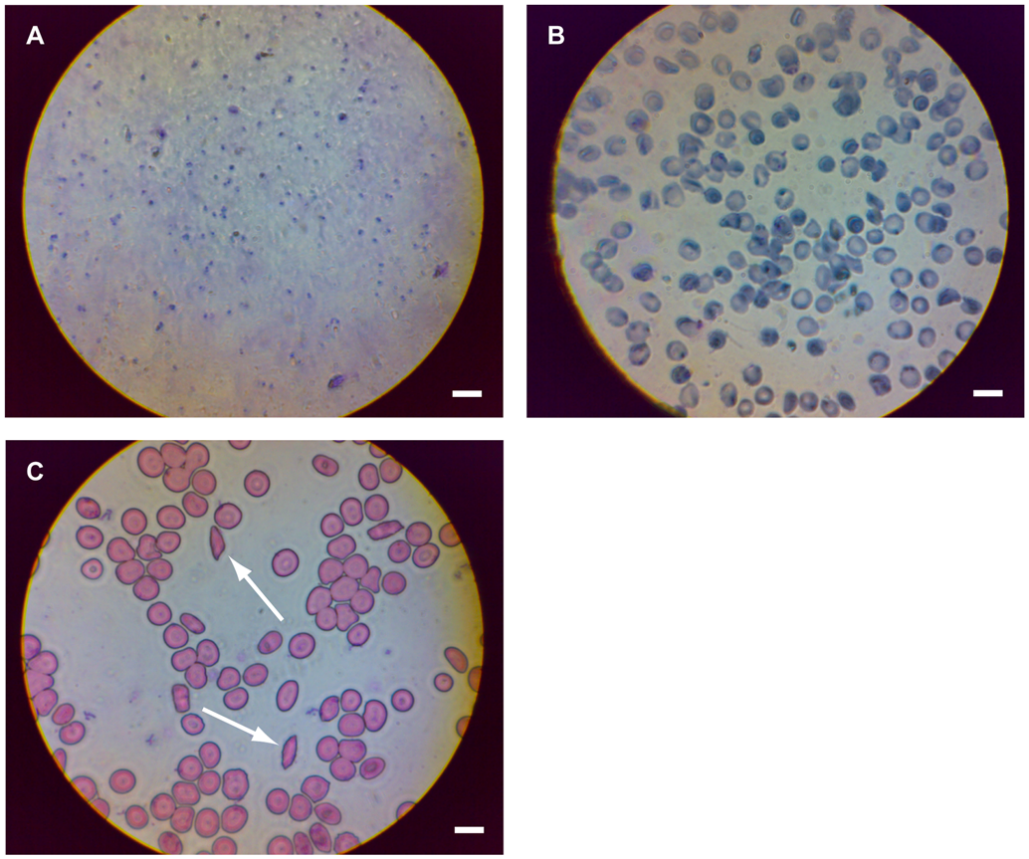
Mobile phone microscopy images of diseased blood smears. (a) Thick smear of Giemsa-stained malaria-infected blood. (b) Thin smear of Giemsa-stained malaria-infected blood. (c) Sickle-cell anaemia blood smear. White arrows point to two sickled red blood cells. Scale bars are 10 µm.

To characterize the mobile phone microscope for clinically relevant applications, we used brightfield illumination to capture high-resolution images of both thin and thick smears of *P. falciparum* malaria-infected blood samples, as well as of sickle cell anemia blood samples (Figure 2).

Malaria is a parasitic disease endemic to many parts of the developing world and is a major global health concern. Diagnosis of malaria is usually performed via observation of parasites in a Giemsa-stained “thick” peripheral blood smear; subsequent speciation is obtained (if desired) from a follow-up examination of a similarly stained thin blood smear at higher magnification and resolution for parasite morphology and species identification [3], [15]. Additionally, it has previously been demonstrated that malaria can be effectively diagnosed from e-mailed smear images [16]. Figures 2a and 2b show color, brightfield images of thick and thin Giemsa-stained smears of malaria-infected red blood cells, respectively, captured on the mobile phone microscope. The quality of the malaria images could be improved by use of a higher NA objective; however, especially for the thick smear (more widely used for screening) the current images are already suggestive of the potential for diagnostic utility.

Sickle cell anemia, another disease that disproportionately affects the developing world, can be diagnosed via blood smears displaying abnormally (sickle) shaped red blood cells (RBCs). Diagnosing and identifying sickle cell patients early in life would enable the implementation of preventive measures to decrease the complication rate and overall disease burden of this life-threatening illness. Our system provides enough image resolution and contrast for the direct observation of sickled cells in blood smears taken from patients with hemoglobin SS disease (Figure 2c), with no additional contrast-enhancing techniques (*e.g.* staining or phase contrast). If needed, however, significant additional contrast can be achieved by the simple expedient of applying an illumination source at an oblique angle to the sample (data not shown). This mobile phone microscopy system could prove to be particularly useful for point-of-care screening of newborns for sickle cell disorders, to identify and treat patients before the onset of symptoms in resource-poor nations, a process already mandatory in the United States and other developed countries [17], [18], [19].

### Fluorescence Imaging of Tuberculosis and Automated Image Analysis

TB is a major world health concern, and treatment entails monitoring of patients over long (6–9 month) periods. While the standard for initial diagnosis is the use of brightfield imaging of a Ziehl-Neelsen stained sputum smear, fluorescent stains are increasing in popularity due to reduced toxicity in preparation, improved ease of reading, and possibly increased accuracy of the resulting diagnosis [2], [20]. Their adoption in the developing world for both diagnosis and monitoring of TB is, however, hindered by a lack of fluorescence microscopy equipment [2], [6], [14] generally due to the cost of the equipment and cost of maintenance.

**Figure 3:**
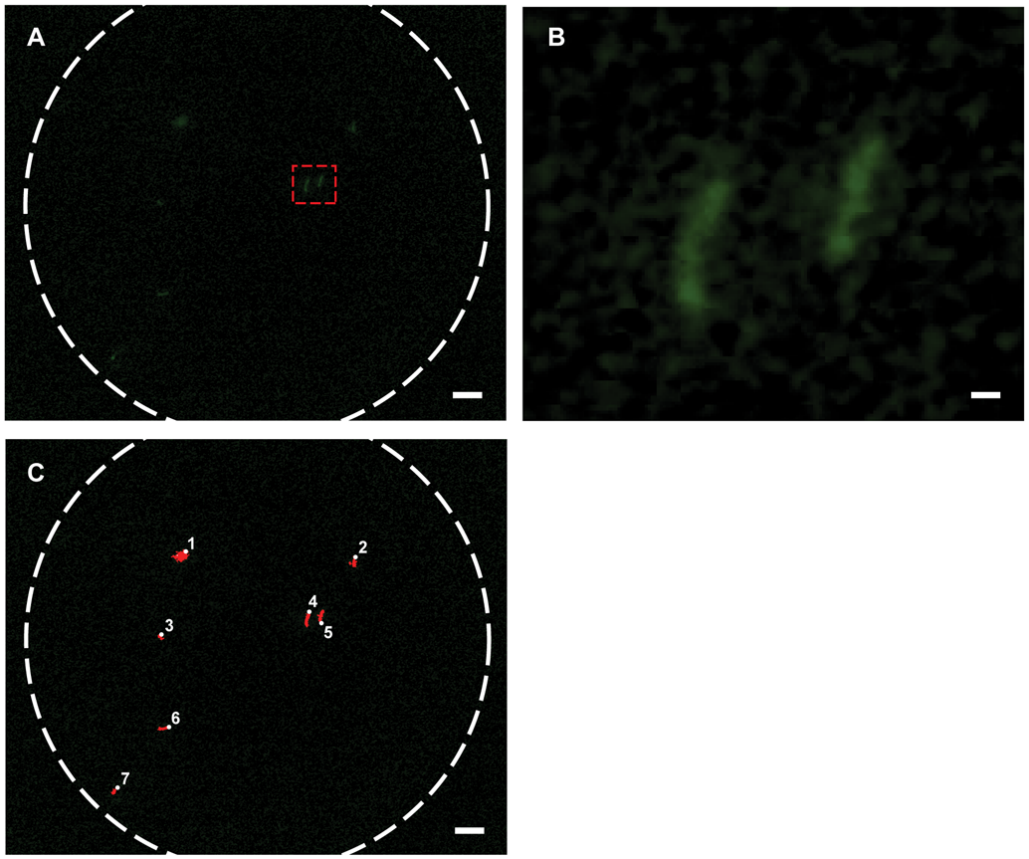
Fluorescence mobile phone microscopy images of tuberculosis in sputum. (a) Fluorescence image of Auramine O-stained TB sputum sample. (b) Enlarged view of two tuberculosis bacilli from red-outlined area in (a). (c) Automated counting of fluorescently-labeled tuberculosis bacilli; counted bacilli are numbered and set to red in the image. Scale bars in (a) and (c) are 10 µm, scale bar in (b) is 1 µm.

Using fluorescence illumination, we were able to capture images of Auramine O-stained *M. tuberculosis*-positive sputum smears (Figure 3a). The resolution of the system was high enough to allow easy identification of individual TB bacteria in the sample, as well as to observe the standard rod-shaped morphology. While we subtracted the background intensity from all images as a matter of course, bacilli were bright enough that background subtraction was not in fact required for reliable identification.

Current standards for the diagnosis of TB using the non-fluorescent Ziehl-Neelsen stain require the screening of upward 100 fields-of-view of ∼180 µm in diameter [21], cumbersome with our system and similarly tedious by eye on a conventional microscope. One of the principal advantages of using the fluorescent Auramine O stain rather than the absorptive Ziehl-Neelsen stain for TB screening is that a lower power (20X) objective may be used [2], with resultantly larger fields of view and thus a reduction in the number of fields (by a factor of 25) which must be examined to cover the same slide area. Such objectives have the added advantage of being less expensive; however, they also have lower light-gathering power making them more challenging to use for fluorescence applications. In our testing we found that a 20X 0.4 NA objective (with a theoretically 5.7X lower light collection efficiency than the 0.85 NA objective) was more than adequate for acquiring images of Auramine O-stained TB bacilli (data not shown). In order to take full advantage of the objective field of view, a sufficient number of detector pixels are required. While our phone had ∼3.2 Megapixels (Mp), camera-phones are well on the way to the ∼4–8 Mp required to image the entire field at maximum resolution.

In addition to the capture and transmission of data, the fact that mobile phones are essentially embedded computer systems offers the opportunity for significant post-processing of images. To demonstrate the diagnostic potential of image processing in this application, we carried out automated bacillus counting of the fluorescent TB images (Figure 3b). For reasons of simplicity we implemented the automated particle count on a laptop computer onto which we had transferred the images, but phone computational resources are sufficient for such tasks to be performed on-phone, providing both an immediate efficiency gain in slide analysis as well as the longer-term potential for automated microbe and pathogen identification.

## Discussion

We have developed a microscope attachment for a camera-enabled mobile phone such that it can be used as a platform for high-resolution clinical light microscopy. The system can reliably capture images of malaria-infected red blood cells from both thin and thick blood smears, as well as images of sickled red blood cells. Additionally, we have demonstrated that mobile phone cameras can be adapted for high-resolution LED-based fluorescent microscopy, using fluorescence imaging of Auramine O-stained sputum smears as a test case.

Microscope-enabled mobile phones have the potential to significantly contribute to the technology available for global healthcare, particularly in the developing world and rural areas where mobile phone infrastructure is already ubiquitous but trained medical personnel, clinical laboratory facilities, and clinical expertise are scarce. By using existing communication infrastructure and expanding the capability of existing mobile phone technology, mobile phone microscopy systems could enable greater access to high-quality health care by allowing rapid, on- or off-site microscopic evaluation of patient samples. As an example, mobile phone microscopy as demonstrated here could provide a rapid, point-of-care method for monitoring TB patients. Such a system would support the World Health Organization's DOTS program, which was established to guide TB eradication efforts by emphasizing, among other factors, the role of quality-assured technology, standardized treatment, and enhanced recording and reporting [4]. With the advent of new 2-minute rapid-staining protocols [22], [23], sample evaluation could potentially be performed in real time while a patient is still in the presence of a healthcare worker, rather than requiring days or weeks. Since we are developing a technology that makes the current and long-standing internationally accepted standards for disease screening in developing countries more portable – rather than creating an entirely new diagnostic assay – we anticipate that a relatively fast time to adoption by clinicians and health workers may be possible.

Not only could such a mobile phone microscopy system help alleviate the problems of inadequate access to clinical microscopy in developing and rural areas, but it would provide those areas remote access to digital record keeping, automated sample analysis, expert diagnosticians, and epidemiological monitoring – the latter enhanced by the ease of location-tagging patient data by cellular triangulation or GPS location data. Combining the mobile phone microscopy system with automated sample preparation systems could address challenges associated with use by minimally-trained health workers and the time involved in imaging multiple fields of view [24]. While future field studies are planned to evaluate the reliability and ease of use of mobile phone microscopy, our present system serves as a proof of principle that clinical imaging of hematologic and infectious diseases is possible with conventional mobile phone camera technology combined with a custom microscopy attachment.

## Materials and Methods

All mobile phones were Nokia N73 camera phones, equipped with a 3.2 megapixel (2048×1536 pixel) CMOS camera with a 5.6×4.2 mm sensor, yielding an ∼2.7 µm pixel spacing. The phone and optical components were mounted using an optical rail system, and laid out as in Figure 1a. A functional, handheld prototype is shown in Figure 1b.

The imaging system consisted of a 20X wide field microscope eyepiece (Model NT39-696, Edmunds Optics) separated by 160 mm from a microscope objective (60X 0.85NA DIN Achromat objective, 160 mm tube length, Model NT38-340, Edmunds Optics). The eyepiece was separated from the camera phone by approximately the focal length of the camera (5.6 mm). For fluorescence imaging, the illumination source was a Luxeon III 455 nm LED (Model LXHL-LR3C, Philips Lumileds) attached to a 3×3 inch microprocessor heat sink with silver conductive epoxy and driven at 700 mA to provide ∼275 mW nominal optical output power. Directly mounted to the LED was a 5° spot lens (OP005, Dialight), followed by a 25.4 mm focal length biconvex lens placed approximately 11 cm from the spot lens and acting as a condenser. Resultant excitation intensity at the sample was 2.0 mW/mm^2^. An excitation filter (D460/50x, Chroma) was placed between the spot and condenser lenses, and an emission interference filter (Chroma D550/50 m) was placed as close as practical to the objective back focal plane. Focus was adjusted by moving the sample position.

Brightfield images were captured using the phone's default camera settings, with the flash disabled. Fluorescent images were captured in the cameras “Night” mode, with the flash disabled. Night mode slightly increases exposure time of the camera to a maximum of 0.2 s, but likely performs software-based contrast adjustments on the image as well. We were not able to manually set the exposure time; however, single images provided adequate signal-to-noise for easy viewing and analysis. For all fluorescent images, we subtracted a background image (captured from a sample area with no fluorescent signal) from the sample image; such subtraction is of low computational overhead and, though we did not do so in these experiments, would be simple to implement in a user-transparent manner as part of the overall image acquisition algorithm. After background subtraction, the JPEG sample image was split into its red-green-blue layers and only the green channel retained. No significant signal was observed in the other channels, despite the demosaicing and JPEG compression implemented on the phone. Images filled a ∼4.8 mm diameter area of the sensor; surrounding blank image areas have been cropped from Figures 1c, 1d, 2, 3a, and 3c for display purposes.

To characterize the resolution of the system, 100 nm fluorescent beads (Fluoresbrite Plain YG Microspheres, Polysciences, Inc.) were diluted 10,000-fold in deionized water and allowed to dry on a 200 line-pair/mm Ronchi ruling. After acquiring a best-focus image of the beads, the emission filter was removed to capture a brightfield image of the Ronchi ruling without refocusing, which we used for calibrating scale (data not shown). We defined resolution as the FWHM of the measured PSF, which in this case was 1.2 µm. This value for resolution should be a slightly conservative estimate since the bead diameter was not deconvolved from the result. The resolution was obtained by averaging the FWHM of seven different beads spread randomly in the field of view. Unfortunately due to lack of information on the phone algorithms for both demoisaicing of the color pixel array and JPEG compression, determining the theoretical system resolution is not possible. The optical magnification of 28X is the product of the 2.7 µm pixel size and 95 nm/pixel scale obtained using the Ronchi ruling. The system field-of-view was measured directly from an image of the Ronchi ruling.

Automated counting of samples performed on a computer using ImageJ [25]. Image threshold was set at three standard deviations above the pixel mean value; bacilli were required to have an area of at least one PSF, 1.57 µm^2^, or 125 pixels, with no upper size limit. While more sophisticated algorithms can be envisioned, the count derived in this manner matched that we performed by eye.

Malaria and sickle cell samples were obtained from patients confirmed to have each disease. TB samples were culture confirmed.

### Ethics Statement

Use of these patient samples was approved by the institutional review board of the University of California, San Francisco. Written informed consent was obtained for all patient samples.
